# *ABCG2* and *NCF4* polymorphisms are associated with clinical outcomes in diffuse large B-cell lymphoma patients treated with R-CHOP

**DOI:** 10.18632/oncotarget.16869

**Published:** 2017-04-06

**Authors:** Duo Liu, Nan Wu, Haiming Sun, Mei Dong, Tianzhu Guo, Peng Chi, Guofu Li, Donglin Sun, Yan Jin

**Affiliations:** ^1^ Department of Medical Genetics, Harbin Medical University, Harbin, Heilongjiang, China; ^2^ Department of Pharmacy, Harbin Medical University Cancer Hospital, Harbin, Heilongjiang, China; ^3^ Department of Pharmacology, School of Basic Medical Sciences, Heilongjiang University of Chinese Medicine, Harbin, Heilongjiang, China; ^4^ Department of Pharmacy, Harbin Medical University-Daqing, Daqing, Heilongjiang, China; ^5^ Department of Neurosurvery, Harbin Medical University Cancer Hospital, Harbin, Heilongjiang, China

**Keywords:** diffuse large B-cell lymphoma, rituximab, pharmacogenetic, prognosis

## Abstract

The impact of pharmacogenetics on predicting survival in diffuse large B-cell lymphoma (DLBCL) remains unclear. We tested 337 DLBCL patients treated with rituximab-cyclophosphamide, doxorubicin, vincristine, and prednisone (R-CHOP) for 9 single nucleotide polymorphisms from 6 genes (*CD20*, *FCGR2A*, *NAD(P)H, ABCC2*, *ABCG2* and *CYP3A5*). Patients who carried the *NCF4* rs1883112 GG genotype showed significantly shorter progression-free survival (PFS) (*P* = 0.023) and event-free survival (EFS) (*P* < 0.001) comparing with A allele. A significantly shortened PFS (*P* = 0.013) and EFS (*P* = 0.002) was also observed in the patients with *ABCG2* rs2231137 GG genotype. Furthermore, the elder (> 60 years old) or male patients with *ABCG2* rs2231137 GG genotype had poorer PFS and EFS than A allele. Moreover, *CD20* rs2070770 CC and *RAC2* rs13058338 AT genotypes were independent predictors of chemotherapy-induced toxicity. Cox proportional hazards analyses demonstrated that the GG genotype of *ABCG2* rs2231137 and *NCF4* rs1883112 were risk factors in DLBCL patients. In conclusion, the identified polymorphisms provide guide for the identification of DLBCL patients who are likely to benefit from chemotherapy.

## INTRODUCTION

Diffuse large B-cell lymphoma (DLBCL) is the most common lymphoid neoplasm, with a wide range of outcomes predicted by the International Prognostic Index (IPI) [1, 2]. Although the introduction of rituximab (R) to the chemotherapy combination of CHOP (cyclophosphamide, doxorubicin, vincristine, and prednisone) has improved prognosis and allowed some tailoring of therapy, a significant fraction of DLBCL patients still fail treatment and die [3–6].

Antibody-dependent cellular cytotoxicity, mediated by effector cells that engage CD20 and the Fc portions of the antibody via receptors (FcγRs) of immunoglobulin, is thought to contribute most to the efficacy of the antibody against malignant cells of B-cell lymphomas [7, 8]. The T allele of CD20 rs2070770 was significantly associated with superior progression-free survival (PFS) [9], while Ding et al. found no significant association [10]. For *FCGR2A*, gene-gene interactions analysis revealed rs1801274 and *IL1RN* rs419598 combination significantly related to adjusted OS of DLBCL in Denmark [11]. And a significant increased NHL risk was observed for the rs1801274 in US [12]. However, no statistically significant difference in survival was found according to rs1801274 allele in Korea [13]. Reactive oxygen species (ROS) involved in cellular functions mediate the therapeutic effects of anticancer drugs. NAD(P)H oxidase is an important endogenous source of ROS, and the small GTPase RAC2 is required for the NAD(P)H oxidase activity [14]. Kim IS et al. observed *ABCG2* rs2231142 polymorphism may correlate with chemotherapy-induced diarrhea in Korean patients [15]. Other host's single nucleotide polymorphisms (SNPs) affecting genes involved in drug metabolism, detoxification and transport may contribute to the prognostic stratification and the predicted toxicity in DLBCL patients [16–23]. However, the impact of genetic polymorphisms on drug activity/metabolism and subsequently treatment outcome in DLBCL patients is largely unclear.

Therefore, in this study we assessed the effects of the nine SNPs in the *CD20*, *FCGR2A*, *NAD(P)H, ABCC2*, *ABCG2* and *CYP3A5* gene on survival in a cohort of 189 Chinese DLBCL patients treated with R-CHOP to define specific subgroups more likely to benefit from therapy.

## RESULTS

### Subjects’ characteristics

A total of 189 patients who received R-CHOP as a frontline therapy for DLBCL were included in the current study. All of them were Chinese Han, including 102 males and 87 females with median age at diagnosis of 55.9 years (range 13–84). Clinical characteristics and treatment outcomes of the cohort at DLBCL diagnosis are displayed in Table 1. The CR and ORR were 149/189 (78.84%) and 152/189 (80.42%), respectively.

**Table 1:** Clinical characteristics of the DLBCL cohort at diagnosis

Clinical characteristics	R-CHOP (n=189)
Age (years)	55.9 (13-84)
Age > 60 years	79/189 (41.80%)
Male: female	102:87
ECOG PS>1	20/189 (10.58%)
B symptom	40/189 (21.16%)
Bulky	58/189 (30.69%)
Extranodal sites>1	36/189 (19.05%)
LDH positive	44/189 (23.28%)
Bone marrow involvement	13/189 (6.88%)
IPI	
0	67/189 (35.45%)
1	65/189 (34.39%)
2	25/189 (13.44%)
3	27/189 (14.29%)
4, 5	5/189 (2.65%)
Ann Arbor stage	
I-II	89/189 (47.09%)
III-IV	100/189 (52.91%)
Subtype	
GCB	62/189 (32.80%)
Non-GCB	127/189 (67.20%)
Response	
CR, n (%)	149/189 (78.84%)
PR, n (%)	3/189 (1.59%)
SD or PD, n (%)	37/189 (19.58%)
ORR (CR+PR), n (%)	152/189 (80.42%)

ECOG PS, Eastern Cooperative Oncology Group performance status; LDH, lactate dehydrogenase; IPI, international prognostic index; GCB, germinal center B cell; CR, complete response; PR, partial response; SD, stable disease; PD, progressive disease; ORR, overall response rate.

### The correlation of clinical characteristics with variant genotypes

We detected the association between the variant genotypes and the clinicopathological data of DLBCL according to ECOG (PS=0 vs. >1), B symptom, Bulky, Extranodal sites (0 vs. >1), LDH level (positive vs. negative), Bone marrow involvement, IPI scores (0 to 5), Ann Arbor stage (I, II vs. III, IV), and Subtype (non-GCB vs. GCB). However, the various SNPs were not correlated with clinical characteristics.

### Response to therapy and outcome

**Figure 1:**
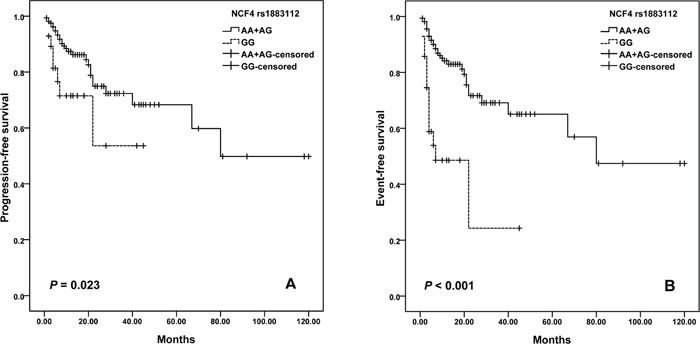
Kaplan–Meier curves illustrate progression-free survival **(A)** and event-free survival **(B)** of patients with R-CHOP chemotherapy according to the variant genotypes of *NCF4* rs1883112.

Patients who carried the rs1883112 GG genotype showed significantly shorter PFS comparing with A allele (log–rank test, *P* = 0.023; Figure 1A). The estimated median PFS for patients who had the GG, and AA plus AG genotypes of rs1883112 was 29.2 months (95% CI =19.7–38.7) and 79.1 (95% CI = 64.4-93.8), respectively. Meanwhile, patients with rs1883112 GG genotype had shorter EFS than patients with AA plus AG genotypes (log–rank test, *P* < 0.001) (Figure 1B).

**Figure 2:**
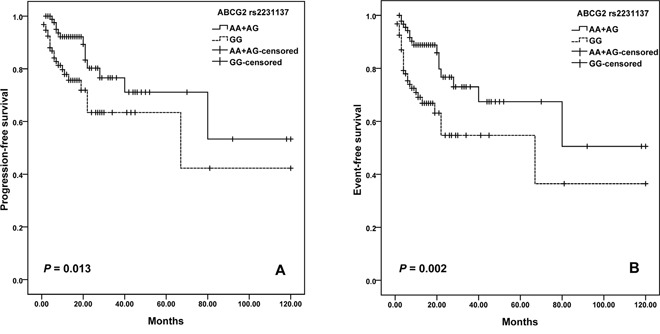
Kaplan–Meier curves illustrate progression-free survival **(A)** and event-free survival **(B)** of patients with R-CHOP chemotherapy according to the variant genotypes of *ABCG2* rs2231137.

**Figure 3:**
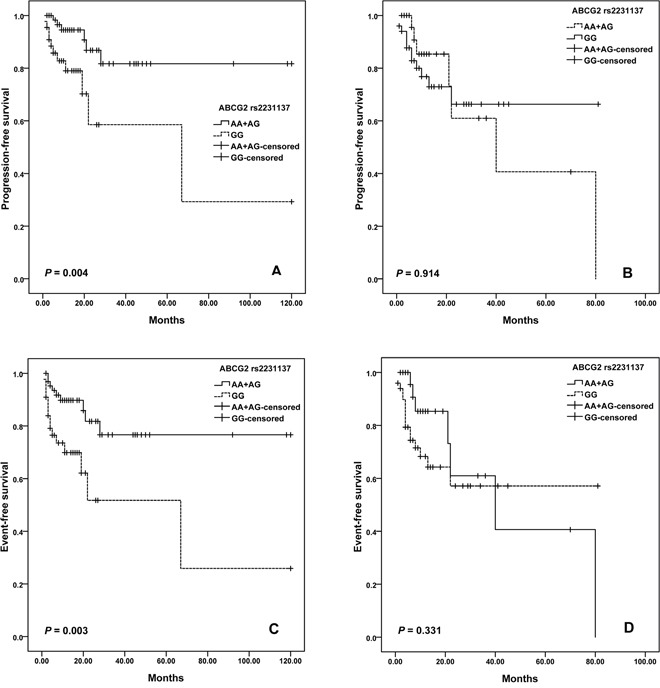
Kaplan–Meier curves illustrate progression-free survival **(A)** and event-free survival **(C)** of elder patients (Age > 60 years), and progression-free survival **(B)** and event-free survival **(D)** of younger patients (Age ≤ 60 years) with R-CHOP according to the variant genotypes of *ABCG2* rs2231137.

**Figure 4:**
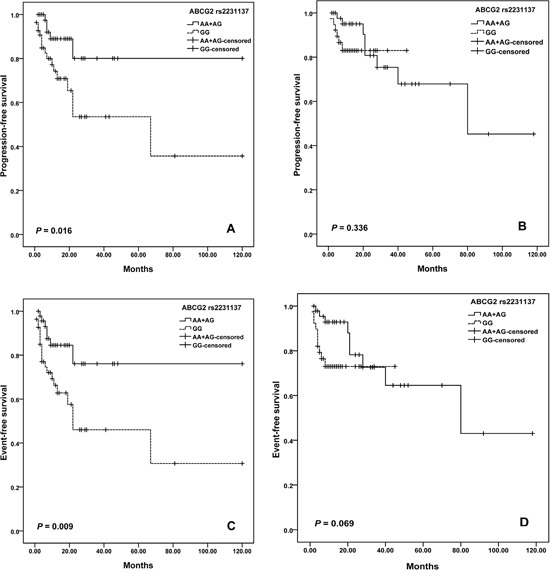
Kaplan–Meier curves illustrate progression-free survival **(A)** and event-free survival **(C)** of male patients, and progression-free survival **(B)** and event-free survival **(D)** of female patients with R-CHOP according to the variant genotypes of *NCF4* rs1883112.

For *ABCG2* variants, we observed a difference in the survival curve for patients. The median PFS was 68.9 months (95% CI = 46.6-91.1) for patients with GG genotype of rs2231137, and was 84.5 months (95% CI =66.3-102.7) months in the AA plus AG genotypes (log-rank; *P*=0.013; Figure 2A). Moreover, patients with rs2231137 GG genotype also had shorter EFS than patients with AA plus AG genotypes (log–rank test, *P =* 0.002) (Figure 2B). Furthermore, potential age/gender implications were investigated. We found that elder patients (>60 years old) with GG genotype of rs2231137 displayed a poor PFS (log–rank test, *P*= 0.004; Figure 3A) and EFS (log–rank test, *P*= 0.003; Figure 3C). Meanwhile, male patients with rs2231137 GG genotype had shorter PFS (log–rank test, *P*= 0.016; Figure 4A) and EFS comparing with A allele (log–rank test, *P*= 0.009; Figure 4C).

With respect to the occurrence of side effects, chemotherapy-induced grade 3 or 4 anemia was less frequent in rs2070770 CC genotype compared with rs2070770 TT (*P*= 0.024; OR=0.201, 95%CI: 0.050–0.807). In addition, we found that rs13058338 AT (*P*= 0.034; OR=2.659, 95%CI: 1.074–6.582) was an independent predictor of grade 3–4 hematologic toxicity (Table 2).

**Table 2:** Association between polymorphisms and grade 3–4 hematological toxicity in DLBCL

Genotypes	*P*^ a^	OR^b^	95% CI
**rs2070770**			
**CC**	**0.024**	**0.201**	**0.050-0.807**
CT	0.087	0.238	0.046-1.229
TT	-	-	
**rs13058338**			
AA	0.807	1.300	0.158-10.677
**AT**	**0.034**	**2.659**	**1.074-6.582**
TT	-	-	

^a^ Comparison of genotype frequencies using Pearson's χ^2^.

^b^ OR and 95% CI values were calculated by logistic regression.

### Multivariate analysis

To estimate the independent impact of each variable on PFS and EFS, a descriptive Cox proportional hazard model was established. The results of the multivariate analysis adjusting for IPI are shown in Table 3. Multivariate Cox regression analysis showed that compared to rs1883112 AA plus AG, the relative risk of GG was 2.410 for PFS (*P* =0.030; HR=2.410, 95% CI: 1.088-5.338), and was 4.464 for EFS (*P* = 0.001; HR=4.464, 95% CI: 2.339-8.520). In addition, the genotype GG of rs2231137 was risk factor for EFS in DLBCL patients (*P* = 0.007; HR=2.315, 95% CI: 1.252–4.281).

**Table 3:** Cox regression analysis of potential factors for PFS and EFS in DLBCL patients adjusting for IPI

Variable		R-CHOP		
		*P* ^a^	Hazard ratio	95% CI
**PFS survival**				
*CD20* (rs2070770)	Dominant mode	0.193	0.584	0.259-1.314
	Recessive mode	0.251	0.311	0.042-2.285
*FCGR2A* (rs1801274)	Dominant mode	0.576	0.799	0.363-1.756
	Recessive mode	0.642	1.172	0.600-2.290
*ABCG2* (rs2231137)	Dominant mode	0.641	1.290	0.442-3.767
	Recessive mode	0.054	1.987	0.989-3.991
*ABCG2* (rs2231142)	Dominant mode	0.123	0.534	0.241-1.184
	Recessive mode	0.982	0.992	0.507-1.943
*NADPH* (rs4673)	Dominant mode	0.785	1.111	0.520-2.376
	Recessive mode	0.983	-	-
*NCF4* (rs1883112)	Dominant mode	0.317	1.437	0.706-2.927
	Recessive mode	**0.030**	**2.410**	**1.088-5.338**
RAC2(rs13058338)	TT vs. AT	0.556	1.247	0.598-2.600
*CYP3A5* (rs776746)	Dominant mode	**0.036**	**0.353**	**0.134-0.935**
	Recessive mode	0.950	1.022	0.519-2.011
**EFS survival**				
*CD20* (rs2070770)	Dominant mode	0.715	0.884	0.455-1.716
	Recessive mode	0.361	0.515	0.124-2.142
*FCGR2A* (rs1801274)	Dominant mode	0.990	0.995	0.481-2.061
	Recessive mode	0.220	1.436	0.805-2.560
*ABCG2* (rs2231137)	Dominant mode	0.644	1.252	0.483-3.249
	Recessive mode	**0.007**	**2.315**	**1.252-4.281**
*ABCG2* (rs2231142)	Dominant mode	**0.041**	**0.479**	**0.236-0.971**
	Recessive mode	0.710	0.894	0.495-1.614
*NADPH* (rs4673)	Dominant mode	0.771	1.103	0.570-2.133
	Recessive mode	0.978	-	-
*NCF4* (rs1883112)	Dominant mode	0.390	1.310	0.707-2.427
	Recessive mode	**0.001**	**4.464**	**2.339-8.520**
*RAC2* (rs13058338)	TT vs. AT	0.918	1.034	0.552-1.937
*CYP3A5* (rs776746)	Dominant mode	**0.007**	**0.324**	**0.142-0.740**
	Recessive mode	0.645	0.871	0.484-1.567

^a^ *P*, HR and 95% CI were assessed using multivariate Cox regression analysis adjusted for IPI.

## DISCUSSION

In this study, we document that: (1) Significantly shortened PFS and EFS after R-CHOP chemotherapy was observed in the patients with NAD(P)H oxidase subunits (*NCF4* rs1883112) GG genotype; (2) ATP-binding cassette transporters (*ABCG2* rs231137) may predict PFS and EFS; (3) Elder (>60 years old) or male patients with GG genotype of rs2231137 displayed poor PFS and EFS comparing with A allele; (4) *NADPH RAC2* rs13058338 (AT) and *CD20* rs2070770 (CC) were independent predictors of chemotherapy-induced toxicity of DLBCL patients.

Rituximab bound to CD20 antigen can be recognized by Fcγ receptor on NK cells and macrophages to mediate killing or phagocytosis of the tumor cells [7]. Studies of neutrophils have shown that *NCF4* encodes the p40phox subunit of the NAD(P)H oxidase which regulates Fcγ receptor-induced superoxide generation following the internalization of phagosomes [26, 27]. Meanwhile, overproduction of ROS involved in neutrophil death upon exposure to chemotherapy [28], and doxorubicin cardiotoxicity [29], and NAD(P)H oxidase is one of the major sources for superoxide generation [30, 31]. Moreover, activation of NCF4 induces ROS-mediated epithelial-mesenchymal transition (EMT) signaling in HeLa cells [32]. Reconstitution of patient B cells with a NCF4 wild-type could restore exogenous Ag presentation and intracellular ROS generation [33].

*NCF4* rs1883112 affects the gene promoter with potential consequences on NCF4 expression and ROS generation [8]. Zhan S et al. observed the *NCF4* that associated with NHL treatment outcome is expressed in hematopoietic cells [34]. Consistent with our findings, Gustafson et al. [20] observed significant association of *NCF4* rs1883112 SNP and PFS. Moreover, *NCF4* rs1883112 was found as an independent predictor against hematologic, infectious and cardiac toxicity for aggressive B-cell non-Hodgkin lymphoma patients [8, 20]. Therefore, *NCF4* rs1883112 G allele is expected to reduce the tumor cytotoxicity of R-CHOP chemotherapy.

*ABCG2* gene located on chromosome 4q22, is a ATP-binding cassette transporter, which increase efflux of doxorubicin, vincristine and prednisone from the cell cytoplasm to the extracellular compartment [35]. ABCG2 is one of the major multidrug resistance (MDR) pumps [36], and targeting *ABCG2* may have therapeutic value in overcoming chemoresistance in DLBCL [37–40]. Heuvel-Eibrink MM et al. found increased expression of ABCG2 was associated with drug resistance in MCF7 cell lines [41].

The *ABCG2* rs2231137 polymorphism results in valine to methionine substitution in the amino acid sequence of the protein. Kondo C et al. observed no significant difference in the levels of protein expression or transport activity of ABCG2 protein between rs2231137 A and G variant in LLC-KP1 cells [42]. While Poonkuzhali B et al. found a significantly lower *ABCG2* mRNA expression was found in liver tissue of Hispanics with the rs2231137 variant genotype than the individuals with wild genotype. It may be due to alternative splicing of mRNA in rs2231137 variant individuals [43]. One study observed a significantly higher risk of grade 3 or more toxicity was found in the patients with rs2231137 A allele compared to the patients with GG genotype [44]. In another study, rs2231137 AG/AA genotypes were found to be independent factors for protecting against infection [21]. In the current study, we observed that DLBCL patients treated with R-CHOP carrying the rs2231137 GG genotype displayed a poorer PFS and EFS compared to patients who carried the AA or GG genotype. Notably, in this study we found that rituximab clearance and efficacy might be gender-biased. Pfreundschuh et al. reported that a better outcome was associated with a slower rituximab clearance and longer exposure times for elderly females but not males [45], while Jaeger et al. suggested that men with a low IPI treated with rituximab had the best outcome [46]. Although no gender or age biased was found for the all patients, we found that elder patients (>60 years old) or male patients with GG genotype of rs2231137 had poorer outcomes comparing with A allele. Further studies are needed for the optimization of rituximab dose and schedule in all subgroups of DLBCL patients.

To date, the prognostic value of *FCGR2A* polymorphism as markers to predict treatment outcome in DLBCL is still being studied [11–13]. Moreover, in rs2070770, the exchange of T to C alleles does not alter the encoded amino acid, so this SNP is not able to change the structure or function of the CD20 protein. It is not yet known the potential role of rs2070770 polymorphism in the susceptibility and prognosis of ritumixab therapy. Rs13058338 TA/AA genotypes were reported to be associated with anthracycline-induced cardiotoxicity in the patients with aggressive CD20+ B-cell lymphoma (mainly diffuse large B cell NHL) in Germany and Italy [8, 21, 22]. Despite the small number of chemotherapy-induced cardiotoxicity in patients, we found that DLBCL patients who carried rs13058338 AT genotype showed a low risk of developing grade 3-4 hematological toxicity. The rs13058338 AT genotype may be a protective factor in DLBCL patients, but further studies are required to confirm the association.

However, the number of patients limited the statistical analysis to demonstrate the relationship between polymorphisms and patient response to chemotherapies. Larger sample sizes and prospective studies, independent collection of clinical outcome and genotyping data, tumor tissue expression analysis, and *in vivo* functional studies are needed to confirm our results and to clearly characterize the underlying mechanisms.

In conclusion, our study suggests that SNPs involved in the host pharmacogenetics of R-CHOP may have a significant role in predicting the failure of therapy and toxicity in patients with DLBCL in the Chinese population. Our findings will help develop a highly cost effective DLBCL treatment based on these predictive and prognostic biomarker tests.

## MATERIALS AND METHODS

### Patient selection

This cohort of patients had newly diagnosed, histopathologically confirmed, and untreated DLBCL. Some blood samples for the genotyping were not available for the patients who were recruited early in the study and these patients were not included in final analysis. Patients without follow-up and those receiving only palliative treatment were excluded. A detailed questionnaire, including requests for demographics information, was administered to all subjects.

At our institution, patients with DLBCL are typically followed and monitored during and after treatments with regularly scheduled clinical and radiographic examinations. Clinicopathological data were collected from medical records including age, gender, Eastern Cooperative Oncology Group (ECOG) performance status, B symptom, bulky, Extranodal sites, LDH level, bone marrow involvement, IPI scores, Ann Arbor stage, and subtype. The molecular classification of DLBCL was analyzed by immunohistochemistry [24]. During treatment the following variables were evaluated: total number of courses per patient, date and type of response and progression, each course of chemotherapy, red blood cell and platelet transfusion and toxicity. Hematological toxicity (anemia, febrile neutropenia, neutrophil count decreased, platelet count decreased, and white blood cell decreased) and extra-hematological toxicity (diarrhea, gastritis, mucositis oral, nausea, vomiting) were graded according to Common Terminology Criteria for Adverse Events of the National Cancer Institute (CTCAE 4.0).

### Treatment protocol

The R-CHOP 21 protocol was identical to the CHOP 21 protocol, except for the addition of intravenous infusion of rituximab (375 mg/m^2^/d) over 4 to 6 h at day 1 before the administration of CHOP drugs. Patients in complete remission received a total of six courses chemotherapy, while patients in partial remission completed eight courses of treatment after four courses. According to Ann Arbor criteria, patients with advanced-stage disease received radiotherapy to bulky sites after chemotherapy. Dose reduction and support treatment were prescribed according to R-CHOP 21 guidelines [5, 6]. The response to therapy was evaluated at the end of the fourth course and at the end of treatment. Follow-up visits were scheduled every 3 months for the first 2 years and every 6 months thereafter.

### Ethic statement

The study was approved by the Ethics Committee of Harbin Medical University with the following reference number, HMUIRB20160004. All participants provided written informed consents and all efforts had been made to protect patient privacy and anonymity. The study was conducted according to the Declaration of Helsinki.

### DNA extraction and genotyping

SNPs were selected according to the following criteria: (1) SNPs known to be relevant for the prediction of outcome or toxicity; (2) SNPs affecting regulatory regions and predicting altered expression or function of the gene; (3) SNPs with a minor allele frequency >5% in the study population. Nine polymorphisms from 6 genes were analyzed as follows: (1) Antibody-dependent cellular cytotoxicity (*CD20* rs2070770, *FCGR2A* rs1801274); (2) *NAD(P)H* oxidase subunits (*NCF4* rs1883112, *RAC2* rs13058338, *CYBA* rs4673); (3) ATP-binding cassette transporters (*ABCC2* rs17222723, *ABCG2* rs2231142, *ABCG2* rs2231137) and (4) Cytochrome P450 enzymes (*CYP3A5* rs776746).

DNA samples were extracted from stored blood samples using QIAamp DNA Blood Midi or Maxi kit (Qiagen, Hamburg, Germany). SNP genotyping was carried out using the Multiplex SNaPshot method. Polymerase chain reactions (PCRs) contained 10–50 ng of DNA, 1×HotStarTaq buffer, 3 mM MgCl_2_, 300 μM of each dNTP, 0.08 μM of each primer, and one unit of HotStar Taq polymerase (Qiagen, Hamburg, Germany) in 20 μl reaction volume. The following touchdown PCR program was used: denaturation at 94°C for 3 min followed by 35 cycles of 94°C for 15 s, annealing at 55°C for 15 s, and extension at 72°C for 30 s. This was followed by 30 cycles of denaturation at 96°C for 10 s, annealing at 52°C for 5 s, and extension at 60°C for 30 s and a final extension at 72°C for 3 min. The PCR products were purified by treatment with Exonuclease I (USB Corporation, Cleveland, Ohio, USA) and Shrimp Alkaline Phosphatase (USB Corporation, Cleveland, Ohio, USA) at 37°C for 15 min followed by incubation at 80°C for 15 min. The extension reaction contained 1×ABI Prism SNaPshot Multiplex ready reaction mix (Applied Biosystems, Grand Island, NY, USA), 0.5 μM of each primer, and 1 μl of each PCR product and was carried out as recommended (Applied Biosystems, Grand Island, NY, USA). The extension PCR products were purified using 1 unit of Shrimp Alkaline Phosphatase and then analyzed using an ABI 3730xl Genetic Analyzer. SNP calling was carried out using GENEMAPPERTM software v.4.0 (Applied Biosystems, Grand Island, NY, USA). For quality control, 15% of the assays were randomly selected for sequencing. The results of the quality control analysis confirmed 100% concordance.

PRIMER5 (http://frodo.wi.mit.edu/) was used to design primers for each SNP within a multiplex. Extension primers were chosen from the sequence up- or down-stream of each SNP immediately, and the primers are listed in Table 4.

**Table 4:** Genotyping primers of SNPs

SNP	Allele	PCR primer	Snapshot primer
*CD20* rs2070770	C/T	F: CACTGCTTCCTTTAGGCATTC	SR: CCTGGGGGGTCTTCTGATGAT
		R: TACCACACAGTCACACAGATG	
*FCGR2A* rs1801274	C/T	F: GTTTCCTGTGCAGTGGTAATC	SR: TTTTTTTGATGGAGAAGGT GGGATCCAAA
		R: TGTGTCTTTCAGAATGGCTGG	
*NCF4* rs1883112	A/G	F: AAATGGAGGCCAGCATTCTAG	SR: AAGGTCACAAGACACCCTGATG
		R: AGGTTTGCAGGAATCCACTTC	
*RAC2* rs13058338	A/G/T	F: GAGAGCAGAGACCATGTTTTC	SR: TTTTTTTTTTTTTTTTTTTTTTTTTTT TTTTTCACCCCCACGGAGGAAGGATGG
		R: ACATCAATCCTTTCTCACCCC	
*CYBA* rs4673	C/T	F: TTTGGTGCTTGTGGGTAAACC	SR: TTTTTTTTTTTTTTTTTTTTTTTTT AGCTTCACCACGGCGGTCATGT
		R: CCCGAACATAGTAATTCCTGG	
*ABCC2* rs17222723	A/T	F: ATCAGGTTTGCCAGTTATCCG	SR: TTTTTTTTTTTTTTTTTTTTTTTTTT CGATTTCTGAAACACAATGAGG
		R: CCAGAGTGAATTTCACACCAC	
*ABCG2* rs2231142	A/C	F: TTGCAGAACTGCAGGTTCATC	SR: TTTTTTTTTTTTTTTCTGAC GGTGAGAGAAAACTTA
		R: TAGTTGTTGCAAGCCGAAGAG	
*ABCG2* rs2231137	A/G	F: AAATGAAGCTGCTCATTGCCG	SR: TTTTTTTTTTAATGTCGAA GTTTTTATCCCA
		R: TTCAGGTCATTGGAAGCTGTC	
*CYP3A5* rs776746	A/G	F: GCACTTGATGATTTACCTGCC	SR: TTTTTTTTTTTTTTTTTTTTTTA AAGAGCTCTTTTGTCTTTCA
		R: AGGGTAATGTGGTCCAAACAG	

PCR, polymerase chain reaction; SNP, single nucleotide polymorphism.

### Statistical analysis

Responses were scored according to the International Working Group criteria [25], and assessed by Fisher exact test and Armitage trend test. Progression-free survival (PFS) was defined as time to disease progression, relapse, or death. Event-free survival (EFS) was defined as the time from the beginning of therapy to disease progression, relapse, death, or initiation of additional (off-protocol) or salvage therapy. The endpoints included complete response (CR), partial response (PR), overall response rate (ORR), stable disease (SD), progressive disease (PD), PFS, and EFS. For each SNP, dominant and recessive genetic models were selected for analysis and the model with highest statistical significance was considered to be the best-fitting model. Survival distributions were estimated with the Kaplan–Meier method and compared with the log-rank test. Multivariate analyses were done using Cox proportional-hazard models to estimate hazard ratios and their 95% confidence interval (CI) for having an event. Analysis for toxicity was performed by Binary-logistic regression analysis with SNP genotypes as the explainable variables.

The statistical analyses were performed using the Statistical Package for Social Sciences (SPSS) version 13.0 statistical software. Statistical significance was set at *P*< 0.05 and all tests were two-sided.

Haiyan fund of Harbin Medical University Cancer Hospital (General Program)(Grant No. JJMS2014-01).
